# Comparison of muscle strength and power in the short physical performance battery for predicting negative outcomes in older adults with mobility limitations

**DOI:** 10.1016/j.jnha.2025.100631

**Published:** 2025-07-15

**Authors:** Hélio José Coelho-Júnior, Alejandro Álvarez-Bustos, Leocadio Rodriguez-Mañas, Francesco Landi, Emanuele Marzetti

**Affiliations:** a Fondazione Policlinico Universitario Agostino Gemelli IRCCS, Rome, Italy; b Department of Geriatrics, Orthopedics and Rheumatology, Università Cattolica del Sacro Cuore, Rome, Italy; c Biomedical Research Center Network for Frailty and Healthy Ageing (CIBERFES), Institute of Health Carlos III, Madrid, Spain; d Department of Geriatrics, Hospital Universitario de Getafe, Madrid, Spain; e Instituto de Investigación IdiPaz, Madrid, Spain

**Keywords:** Sarcopenia, Physical function, Physical performance, Disability, Hospitalization

## Abstract

**Objectives:**

The present study examined sex-specific associations between Short Physical Performance Battery (SPPB) indexes operationalized according to lower limb muscle strength and power and the occurrence of negative events in older adults with mobility limitations.

**Design:**

Secondary longitudinal analysis of a randomized controlled trial

**Setting:**

Community-Dwellers.

**Participants:**

Older adults (70+ years) with mobility limitations.

**Methods:**

Participants completed the SPPB under standardized conditions. Muscle power measures were estimated according to results of the 5-time sit-to-stand (5STS) test using validated equations. Then, four SPPB indexes were created by replacing 5STS test results with muscle power measures. Outcomes were assessed after 24 months of randomization and included mobility disability, hospitalization, and death.

**Results:**

Data from 1,422 participants were analyzed. Cox regression models revealed sex-specific associations between SPPB variants and adverse outcomes. SPPB indexes incorporating muscle strength or power were significantly associated with mobility disability in both sexes, and with hospitalization and death in women. According to Akaike’s Information Criterion, muscle power-based SPPB indexes provided the best fit for predicting negative outcomes.

**Conclusions:**

Sex-specific associations exist between modified SPPB indexes and adverse outcomes in older adults with mobility limitations. Notably, SPPB variants incorporating muscle power measures demonstrated the best fit for adverse outcomes in comparison to traditional SPPB.

**Trial registration:**

ClinicalTrials.gov identifier: NCT02582138 (registration date: 2015-10-08).

## Highlights

• Sex-specific associations are observed between SPPB indexes and adverse outcomes.
• SPPB indexes are associated with disability in men.
• SPPB indexes are associated with disability, hospitalization and death in women.
• SPPB using muscle power shows the strongest links to negative outcomes.
• SPPB indexes are good predictors of disability and hospitalization.

## 1 Introduction

The preservation of physical performance plays a crucial role in promoting successful aging, as it directly impacts the capacity of older adults to maintain independence [1]. On the other hand, declines in physical function contribute to the development of chronic conditions (e.g., sarcopenia) and geriatric syndromes (e.g., frailty) [[2], [3], [4]], in addition to being associated with the occurrence of numerous health negative events, including falls, hospitalizations, institutionalization, and death [5,6]. As such, monitoring variations in physical performance is pivotal in effectively managing the health status of older adults [3,4].

The Short Physical Performance Battery (SPPB) was proposed by Guralnik et al. [7] in 1994 as a practical, efficient, and safe method to assess lower extremity physical performance in older adults. The battery involves the assessment of balance, walking speed, and lower limb muscle strength. At the end of each test, a score from 0 to 4 points is attributed according to the test person’s performance, with a maximum score of 12, which represents better physical function. SPPB was originally validated against the risk of institutionalization and death [7], and subsequent studies have expanded these results to other negative outcomes, such as falls [8], mobility disability [9], and cardiovascular events [10]. These results led experts in the field to recognize the SPPB as a valid instrument for monitoring sarcopenia [3] and frailty [11,12].

Muscle strength is a major component of the SPPB, as loss of muscle strength is a main consequence of muscle aging [[13], [14], [15]] and is significantly associated with the occurrence of adverse outcomes [[16], [17], [18], [19]]. However, muscle power, the capacity to generate muscle strength rapidly [20], declines earlier and to a greater extent than muscle strength [20], with a steeper decline in women [21,22].

Furthermore, investigations have found that muscle power might be a better predictor of physical independence, functional performance, mobility, and death than muscle strength [16,23,24]. These findings differ according to the anthropometric parameters (e.g., body mass, appendicular skeletal muscle) used for adjustment, suggesting that associations with health parameters might be dependent on the type of muscle power parameter analyzed and calling for a deep exploration of the various muscle power measures available.

Because impairments in muscle power might be a more relevant proxy of muscle failure [25] than those in muscle strength, it may be hypothesized that replacing muscle strength with muscle power in the SPPB might enhance its ability to predict negative outcomes. To explore this hypothesis, the current study examined data of the Sarcopenia and Physical fRailty IN older people: multi-componenT Treatment strategies (SPRINTT) trial to compare the capacity of various SPPB indexes operationalized according to lower limb muscle strength and power to predict mobility disability, hospitalization, and death in older men and women with mobility limitations.

## 2 Material and methods

### 2.1 Study design

This study is a secondary longitudinal analysis of the SPRINTT clinical trial (ClinicalTrials.gov identifier: NCT02582138 [registration date: 2015-10-08]) [26]. SPRINTT was a multicenter, evaluator-blinded, randomized controlled trial (RCT) that tested whether a multicomponent intervention, encompassing physical activity with technological support and nutritional counseling, would reduce the risk of mobility disability in older adults with mobility limitations compared with a healthy aging lifestyle educational program. Details on the RCT methodology and main findings are reported elsewhere [26,27]. The ethics committee of the Università Cattolica del Sacro Cuore (Rome, Italy; coordinating center) granted approval to the study protocol (protocol # 15611/15). The protocol was then ratified by the ethics committees of all participating institutions. The study was conducted in accordance with the ethical principles outlined in the Declaration of Helsinki, originally adopted in 1964 and later amendments, ensuring the protection of the rights, well-being, and dignity of all participants. All candidate participants provided written informed consent before being evaluated for inclusion.

### 2.2 Participants

Trial participants were men and women aged 70 years or older with mobility limitations (SPPB scores between 3 and 9). Participants were included if they had: (a) scores ranging from 3 to 9 points on the SPPB [28]; (b) low appendicular lean mass (ALM), either absolute or body mass index (BMI)-adjusted, according to sex-specific cut-points [29]; and (c) no mobility disability, operationalized as being able to complete a 400-m walk test in less than 15 min without sitting, stopping for more than one min, help of another person, or using a walker [30]. Main exclusion criteria were self-reported walking disability (i.e., inability to walk across a small room or 400 m without the help of another person, a walker, a tripod, or quad cane), cognitive impairment (defined as a mini-mental state examination (MMSE) [31], score <24/30), presence of a terminal illness, participation in a structured physical activity program, contraindications to safely engage in trial activities as judged by local study physicians, and plans to relocate out of the study area within at least two years. For the present study, participants with missing data for the 5-time sit-to-stand (5STS) test were excluded from the analysis (n = 97).

### 2.3 Short physical performance battery indexes

The SPPB was performed under standardized conditions [7]. The battery involves three tests that evaluate lower-body function: a hierarchical test of standing balance, a 4-m walk speed test, and the 5STS. Each SPPB component test is scored from 0 to 4, with a score of 0 representing the inability to perform the test and a score of 4 representing the highest category of performance. For the balance tasks, participants are asked to stand with their feet side by side, followed by the semitandem (heel of one foot alongside the big toe of the other foot) and tandem (heel of one foot directly in front of and touching the other foot) positions for 10 s each. For gait speed, a 4-m walk speed test at the participant’s usual pace was timed. For the 5STS, participants were requested to rise from a chair five times as fast as possible with their arms crossed in front of the body. A summary score was obtained by adding the scores of each individual SPPB component (range 0–12), with higher scores indicating better lower-body function.

Results of the 5STS test were used to estimate absolute (AMP), relative (RMP), allometric (ALMP), and specific muscle power (SMP) measures according to the equations proposed by Alcazar et al. [32]:

(1) AMP (W) = Body mass kg × 0.9 × g ×[stature (m) × 0.5 - chair height (m)][5STS test time (s)no. of STS repetitions] × 0.5

(2) RMP (W/kg) = Absolute muscle power (W)Body mass (kg)

(3) ALMP (W/m^2^) = Absolute muscle power (W)Stature (m2)

(4) SMP (W/kg) = Absolute muscle power (W)Appendicular Lean Muscle (kg)

Then, 5STS results of the “traditional” SPPB (SPPBt) were replaced with muscle power parameters using sex-specific quartiles (appendix 1). This approach allowed development of four SPPB indexes: (i) AMP (SPPBamp), (ii) RMP (SPPBrmp), (iii) ALMP (SPPBalmp), and iv) SMP (SPPBsmp).

### 2.4 Outcomes

Mobility disability was operationalized as the inability to complete the 400-m walk test in less than 15 min without sitting, stopping for more than one min, and without the help of another person or a walker [30]. For the test, participants were asked to complete 10 laps around a 20-m course at their usual pace without overexerting themselves. The 400-m walk test was administered in the same place, selected by each of the 16 clinical sites, at baseline, at three months, and every six months thereafter for up to two years following randomization. Mobility disability was considered ascertained at the date the participant: (a) failed the 400-m walk test, (b) attended a scheduled visit and was found unable to attempt the test, or (c) died. A rigorous procedure was devised for outcome adjudication in cases when the 400-m walk test was not performed. Data on hospitalizations were collected at follow-up visits through self-report. Information on deaths was obtained through proxy-report, hospital records, and national registries.

### 2.5 Covariables and adjusted variables

Body mass and stature were measured through a stadiometer and an analogic medical scale, respectively. BMI was calculated as the ratio between body mass (kg) and the square of stature (m^2^). ALM was measured by whole-body dual-energy X-ray absorptiometry (DEXA) on a Hologic (Bedford, MA, USA) or a GE Lunar (General Electric, Boston, MA, USA) scanner. The MMSE [31] was used to assess global cognitive function. The Center for Epidemiological Studies Depression Scale (CES-D) [9], a 20-item self-rating questionnaire, was employed as a screening tool for depressive symptoms. The Mini Nutritional Assessment (MNA) was used to estimate the risk of malnutrition. Accordingly, participants were categorized into well-nourished, at risk of malnutrition, and malnourished categories. Multimorbidity [33] was operationalized as the co-existence of three or more of the following conditions obtained through self-report and medical records: type-2 diabetes mellitus, cardiovascular disease, osteoarthritis, and cancer. Covariates included in the regression models were selected based on their established associations with the adverse health outcomes examined in the present study [[34], [35], [36], [37], [38], [39], [40], [41], [42]], in order to reduce residual confounding and improve model validity.

### 2.6 Statistical analysis

Data of 1422 participants were analyzed. Those who had missing data for the 5STS were excluded from the analysis. All participants had complete data for each outcome measure (i.e., disability, hospitalization, and death), resulting in no missing data for these variables. Eight participants did not complete the CES-D, and seven had incomplete data for the MNA. Consequently, these participants were excluded from the adjusted analysis using listwise deletion. Data are presented as mean ± standard deviation (SD) for continuous variables, and frequency (percentages) for categorical variables. Analyses were stratified by sex. Kaplan–Meier and Cox proportional hazards analyses were used to identify predictors of survival. These analyses were conducted using the specific time when the event of interest occurred (e.g., 136 days, 247 days, 723 days), which varied for each participant. The final models were adjusted for age, BMI, MMSE, CES-D, MNA, multimorbidity, and randomization arm. Akaike’s Information Criterion (AIC) was calculated to assess the model fit and determine the best-fitting model among those tested. This statistical approach was used instead of other methods for evaluating models (e.g., Likelihood Ratio Test [LRT]), given that AIC allows comparison of multiple models simultaneously, in addition to considering both fit and complexity. In contrast, the use of LRT, for example, is limited to the comparison of two models at a time, and focuses solely on whether the additional parameters in the complex model significantly improve the fit. Delta (Δ) AIC was calculated by subtracting the AIC of the best-fitting model (lowest AIC [LAIC]) from the AIC of each model being compared. Receiver operating characteristic (ROC) curve analyses were used to evaluate the diagnostic accuracy of SPPB indexes through adjusted models. In this analysis, the “time” variable refers to a specific time point and is the same for all participants (e.g., 2 years). Area under the ROC curve (AUC) was categorized as acceptable (0.7–0.8), excellent (0.8–0.9) or outstanding (>0.9). Effect sizes and 95% confidence intervals (95% CIs) were calculated for all analyses, and the level of significance was set at P < 0.05. All analyses were performed using SPSS statistical package (version 23; IBM, New York, NY, USA).

## 3 Results

### 3.1 Sample characteristics

The main characteristics of study participants (n = 1422) according to sex are shown in Table 1. Mean 5STS values (men = 19.1 ± 6.7 s; women = 19.3 ± 6.7 s) were lower than cutoff points for sarcopenia according to the revised consensus of the European Working Group on Sarcopenia in Older People (EWGOSP) [3]. RMP was lower than cutoff points proposed by Alcazar et al. [43] to identify individuals with poor mobility. Men and women had a median overall SPPB score of 7, ranging from 3 to 9, as per protocol. The incidence of disability, hospitalization, and death was 7.8%, 38.6%, and 1.3% in men, and 9.0%, 10.1%, 30.1%, and 0.7% in women, respectively.

**Table 1:** Main characteristics of study participants.

Variables	Total (n = 1422)	Men (n = 396)	Women (n = 1026)	P-value
Age, years	79.0 ± 5.8	79.5 ± 6.2	78.7 ± 5.7	0.026
BMI, kg/m^2^	28.5 ± 5.7	29.2 ± 5.2	28.3 ± 5.9	0.005
SPPB*, score	7 (3−9)	7 (3−9)	7 (3−9)	0.999
5STS, s	19.2 ± 6.7	19.1 ± 6.7	19.3 ± 6.7	0.614
AMP, W	119.4 ± 50.2	157.7 ± 58.1	104.6 ± 37.4	<0.001
RMP, W/kg	1.7 ± 0.4	1.9 ± 0.5	1.6 ± 0.4	<0.001
ALMP, W/m^2^	47.7 ± 17.2	56.3 ± 19.0	44.3 ± 15.2	<0.001
SMP, kg	7.2 ± 2.2	7.4 ± 2.3	7.1 ± 2.2	0.0260
IHG, kg	20.2 ± 8.6	28.6 ± 9.2	16.9 ± 5.6	<0.001
ALM, kg	16.5 ± 3.8	21.1 ± 3.5	14.7 ± 2.2	<0.001
Death, n (%)	61 (4.3)	25 (6.3)	36 (3.5)	—
Time to death, days	799.5 ± 276.6	769.5 ± 270.6	811.1 ± 278.1	0.010
Hospitalization, n (%)	461 (32.4)	153 (38.6)	308 (30.0)	—
Time to hospitalization, days	657.6 ± 340.6	610.1 ± 335.9	675.9 ± 340.8	0.001
Mobility Disability, n (%)	633 (44.5)	192 (48.5)	441 (43.0)	—
Time to mobility disability, days	593.9 ± 363.3	546.5 ± 350.0	612.2 ± 366.8	0.001
MMSE, score	28.0 ± 1.8	28.0 ± 1.7	28.0 ± 1.8	1.000
CES-D, points	5.6 ± 4.0	4.6 ± 3.3	6.1 ± 4.1	<0.001
MNA, points	12.7 ± 1.7	13.0 ± 1.6	12.6 ± 1.7	<0.001

Values are shown as mean ± standard deviation. * Values shown as median (min−max). 5STS = 5-sit to stand test;

ALM = Appendicular lean mass; ALMP = Allometric muscle power; AMP = Absolute muscle power; BMI = Body mass index; CES-D = Center for Epidemiological Studies Depression Scale; IHG = Isometric handgrip test; MMSE = Mini mental state examination; MNA = Mini nutritional statement. RMP = Relative muscle power; SMP = Specific muscle power; SPPB = Short Physical Performance Battery.

When participant characteristics were compared by sex, men were older and had higher BMI, ALM, muscle power, and strength. In contrast, women had longer times to death, hospitalization, and disability, higher CES-D scores, and lower MNA scores.

### 3.2 Associations between SPPB indexes and negative outcomes in men

Unadjusted and adjusted Cox regression analyses for the associations between SPPB indexes and negative outcomes in men are shown in Table 2. In the unadjusted analysis, all SPPB indexes were significantly associated with both mobility disability and hospitalization. In contrast, only SPPBalmp was significantly associated with death. After adjustment for possible confounders, all SPPB indexes remained significantly associated with disability. However, no significant associations were observed between any SPBB indexes and either hospitalization or death.

**Table 2:** Unadjusted and adjusted Cox regressions for the predictive capacity of SPPB indexes toward negative outcomes in men.

	Unadjusted		Adjusted*	
Variables	HR	P-value	HR	P-value
*Mobility Disability*				
SPPBt	0.722 (0.655, 0.795)	**<0.001**	0.735 (0.665, 0.812)	**<0.001**
SPPBamp	0.768 (0.702, 0.840)	**<0.001**	0.782 (0.711, 0.859)	**<0.001**
SPPBrmp	0.765 (0.697, 0.840)	**<0.001**	0.776 (0.707, 0.852)	**<0.001**
SPPBalmp	0.768 (0.700, 0.842)	**<0.001**	0.780 (0.708, 0.860)	**<0.001**
SPPBsmp	0.790 (0.718, 0.868)	**<0.001**	0.807 (0.733, 0.888)	**<0.001**
*Hospitalization*				
SPPBt	0.863 (0.772, 0.964)	**0.009**	0.909 (0.809, 1.021)	0.108
SPPBamp	0.876 (0.796, 0.964)	**0.007**	0.922 (0.829, 1.025)	0.133
SPPBrmp	0.873 (0.788, 0.968)	**0.010**	0.914 0.823, 1.016)	0.096
SPPBalmp	0.866 (0.785, 0.955)	**0.004**	0.906 (0.813, 1.011)	0.078
SPPBsmp	0.885 (0.797, 0.983)	**0.023**	0.930 (0.834, 1.037)	0.193
*Death*				
SPPBt	0.780 (0.606, 1.005)	0.054	0.823 (0.624, 1.084)	0.166
SPPBamp	0.812 (0.645, 1.021)	0.075	0.845 (0.649, 1.101)	0.212
SPPBrmp	0.855 (0.670, 1.092)	0.210	0.860 (0.667, 1.110)	0.247
SPPBalmp	0.775 (0.612, 0.982)	**0.035**	0.802 (0.611, 1.054)	0.114
SPPBsmp	0.838 (0.654, 1.072)	0.159	0.861 (0.662, 1.121)	0.267

Bold denotes statistical significance.

ALMP = Allometric muscle power; AMP = Absolute muscle power; CI = Confidence interval; HR= Hazard ratio; RMP = Relative muscle power; SMP = Specific muscle power; SPPB = Short Physical Performance Battery; T = Traditional.

* Adjusted for age, body mass index, risk of malnutrition, depressive symptomatology, global cognitive function, multimorbidity, and randomization arm.

### 3.3 Associations between SPPB indexes and negative outcomes in women

Unadjusted and adjusted Cox regression analyses for the associations between SPPB indexes and negative outcomes in women are shown in Table 3. In the unadjusted analysis, all SPPB indexes were significantly associated with disability conditions, hospitalization, and death. These associations remained significant after adjustment for potential confounding variables.

**Table 3:** Unadjusted and adjusted Cox regressions for the predictive capacity of SPPB indexes toward negative outcomes in women.

	Unadjusted		Adjusted*	
Variables	HR (95% CI)	P-value	HR HR (95% CI)	P-value
*Mobility Disability*				
SPPBt	0.712 (0.669, 0.758)	**<0.001**	0.759 (0.712, 0.810)	**<0.001**
SPPBamp	0.803 (0.760, 0.849)	**<0.001**	0.817 (0.772, 0.865)	**<0.001**
SPPBrmp	0.758 (0.718, 0.801)	**<0.001**	0.801 (0.756, 0.848)	**<0.001**
SPPBalmp	0.795 (0.752, 0.841)	**<0.001**	0.804 (0.760, 0.851)	**<0.001**
SPPBsmp	0.777 (0.736, 0.821)	**<0.001**	0.809 (0.766, 0.856)	**<0.001**
*Hospitalization*				
SPPBt	0.840 (0.779, 0.905)	**<0.001**	0.864 (0.779, 0.934)	**<0.001**
SPPBamp	0.894 (0.837, 0.954)	**<0.001**	0.921 (0.859, 0.988)	**0.021**
SPPBrmp	0.885 (0.828, 0.945)	**<0.001**	0.909 (0.848, 0.974)	**0.007**
SPPBalmp	0.884 (0.827, 0.944)	**<0.001**	0.908 (0.846, 0.974)	**0.007**
SPPBsmp	0.877 (0.822, 0.936)	**<0.001**	0.904 (0.844, 0.968)	**0.004**
*Death*				
SPPBt	0.652 (0.531, 0.801)	**<0.001**	0.716 (0.577, 0.889)	**0.002**
SPPBamp	0.691 (0.573, 0.832)	**<0.001**	0.774 (0.634, 0.945)	**0.012**
SPPBrmp	0.704 (0.587, 0.845)	**<0.001**	0.763 (0.627, 0.928)	**0.007**
SPPBalmp	0.700 (0.579, 0.845)	**<0.001**	0.785 (0.643, 0.958)	**0.017**
SPPBsmp	0.703 (0.586, 0.843)	**<0.001**	0.777 (0.641, 0.943)	**0.011**

Bold denotes statistical significance.

ALMP = Allometric muscle power; AMP = Absolute muscle power; CI = Confidence interval; HR= Hazard ratio; RMP = Relative muscle power; SMP = Specific muscle power; SPPB = Short Physical Performance Battery; T = Traditional.

* Adjusted for age, body mass index, risk of malnutrition, depressive symptomatology, global cognitive function, multimorbidity, and randomization arm.

### 3.4 Comparisons between SPPB indexes

AICs for comparisons among SPPB indexes are shown in Table 4. In people from both sexes, SPPBrmp exhibited the lowest AIC for mobility disability. SPPBrmp also exhibited the lowest AIC for death, whereas SPPBsmp had the lowest value for hospitalization.

**Table 4:** AICs for comparisons among SPPB indexes.

Variables	Mobility Disability	Hospitalization	Death
	Men		
SPPBt	2108.0 (ΔAIC = 145.0)	–	–
SPPBamp	1965.3 (ΔAIC = 2.3)	–	–
SPPBrmp	1963.0 (LAIC = 0)	–	–
SPPBalmp	1966.7 (ΔAIC = 3.7)	–	–
SPPBsmp	1971.7 (ΔAIC = 8.7)	–	–
	Women		
SPPBt	5682.8 (ΔAIC = 292.7)	4164.1 (ΔAIC = 232.6)	464.2 (ΔAIC = 10.7)
SPPBamp	5398.1 (ΔAIC = 8.0)	3930.8 (ΔAIC = 2.9)	454.7 (ΔAIC = 1.2)
SPPBrmp	5390.1 (ΔAIC = 0)	3929.0 (ΔAIC = 1.1)	453.5 (ΔAIC = 0)
SPPBalmp	5390.9 (ΔAIC = 0.8)	3929.0 (ΔAIC = 1.1)	455.1 (ΔAIC = 1.6)
SPPBsmp	5392.0 (ΔAIC = 1.9)	3927.9 (ΔAIC = 0)	454.2 (ΔAIC = 0.7)

ALMP = Allometric muscle power; AMP = Absolute muscle power; HR= Hazard ratio; RMP = Relative muscle power; SMP = Specific muscle power; SPPB = Short Physical Performance Battery; T = Traditional.

ΔAIC ≤ 2 suggests models are equally plausible; ΔAIC between 4–7 indicates a moderate difference; ΔAIC > 10 indicates a strong difference in model fit.

### 3.5 Ability to discriminate individuals at higher risk of negative events

ROC curve results are shown in Table 5. All SPPB indexes exhibited acceptable AUC results for predicting mobility disability and death in both men and women. In contrast, SPPB indexes had a poor capacity to predict hospitalization.

**Table 5:** Predictive capacity of SPPB indexes adjusted for covariates toward negative outcomes.

Variables	AUC	
	Men	Women
*Mobility Disability*		
SPPBt	0.719	0.714
SPPBamp	0.709	0.708
SPPBrmp	0.709	0.713
SPPBalmp	0.708	0.711
SPPBsmp	0.701	0.712
*Hospitalization*		
SPPBt	0.624	0.606
SPPBamp	0.620	0.600
SPPBrmp	0.623	0.604
SPPBalmp	0.623	0.603
SPPBsmp	0.616	0.607
*Death*		
SPPBt	0.758	0.725
SPPBamp	0.766	0.718
SPPBrmp	0.765	0.725
SPPBalmp	0.775	0.713
SPPBsmp	0.765	0.718

*ROC curves adjusted for age, body mass index, mini-mental state examination, center for epidemiological studies depression scale, mini nutritional assessment, multimorbidity, and randomization arm. ALMP = Allometric muscle power; AMP = Absolute muscle power; AUC = Area under the curve; HR= Hazard ratio; RMP = Relative muscle power; SMP = Specific muscle power; SPPB = Short Physical Performance Battery; T = Traditional.

## 4 Discussion

The main findings of the present study indicate that SPPB indexes are significantly associated with negative outcomes in older adults with mobility limitations. Indeed, all SPPB indexes were significantly associated with mobility disability in both sexes. However, sex-specific associations were also observed, as SPPB indexes were significantly associated with hospitalization and death in women, but not in men. When comparisons among indexes were made to identify the best-fitting model, AIC results indicated that, among the models tested, SPPBrmp provided the best fit for predicting mobility disability in both sexes and for death in women. In contrast, the SPPBsmp model showed the best fit for predicting hospitalization in women. AUC results suggest that SPPB indexes were effective in identifying older adults with mobility limitations at higher risk of experiencing disability and death, but not hospitalization.

To the best of our knowledge, this is the first study that examined associations between SPPB indexes operationalized according to muscle strength or power and negative outcomes in older adults. Our results are aligned with previous studies [9,44,45] by indicating that SPPB, regardless of the operationalization model, is a good predictor of negative events, reinforcing the importance of this instrument to assess overall physical performance in older adults. Nonetheless, our findings suggest that the associations between SPPB indexes and adverse outcomes may be more pronounced in women compared to men.

The observation that SPPB indexes were significantly associated with mobility disability in both sexes likely reflects the fact that it is a more direct outcome of impaired lower limb function. However, hospitalization and death are more complex outcomes influenced by multiple factors beyond physical performance alone. This interpretation is further reinforced by the finding that the associations between SPBB indexes and adverse outcomes (i.e., hospitalization and death) were no longer significant after adjustment for covariates.

Women typically experience a more pronounced decline in the capacity to produce strength and power with aging [21,22], making them more susceptible to frailty [46] and its associated undesirable health outcomes, such as hospitalization, institutionalization, and death [47,48]. Furthermore, age-related changes in body composition appear to have more severe consequences on physical function in women [49], potentially serving as a key link between declines in neuromuscular performance and the onset of adverse outcomes.

Indeed, women experience significant increases in body fat and visceral adipose tissue with aging [[50], [51], [52]]. These gains in fat mass are further accelerated during menopause transition [50] and are often accompanied by an ectopic fat deposition within the muscle tissue, a condition known as myosteatosis [53]. Myosteatosis is more frequently observed in women [53] and has been shown to be negatively associated with muscle activation during maximal contractions [54,55], accounting for up to 36% of the variance in neuromuscular activation among older adults [56].

Notably, the adipose tissue is increasingly recognized as a metabolically active endocrine organ that produces adipokines, which regulate energy metabolism, satiety, inflammation, and insulin sensitivity [57,58]. These molecules contribute to conditions like atherosclerosis, hypertension, type 2 diabetes, and frailty, ultimately increasing the risk of adverse outcomes such as hospitalization and death [57,58].

A complementary explanation is based on the fact that men and women often display distinct patterns of leisure-time physical activity and domestic work [59]. Men tend to be more active [60,61], engaging in more vigorous activities [60] that recruit type II muscle fibers, crucial for force and power, helping maintain or improve physical performance [62]. Additionally, high-intensity activity is linked to a lower risk of cardiovascular and metabolic diseases [63].

Our results indicate that substituting 5STS performance with muscle power measures strengthened the associations with adverse outcomes. Numerous studies have found that RMP and SMP are significantly associated with the occurrence of negative events (i.e., mobility problems, hospitalization, and death), in older adults [[64], [65], [66], [67]]. Furthermore, other investigations have identified sex-specific associations between muscle power measures and functionality in older adults [60,69].

These findings highlight muscle power as a crucial parameter in physical evaluation and suggest that adjusting results for by the weight lifted (RMP) and how much of this amount is functional (SMP) strengthens SPPB associations with adverse outcomes. However, a closer look at the AIC results reveals that differences between the various SPPB muscle power indexes are minimal, about 0.25% (e.g., ΔAIC = 8.7 between SPPBrmp and SPPBsmp) in men and 0.05% in women for hospitalization (e.g., ΔAIC = 2.9 between SPPBrmp and SPPBamp), while differences between these indexes and SPPBt were somewhat larger (up to 7% in men [ΔAIC = 145.0 between SPPBrmp and SPPBt] and 5% in women [ΔAIC = 292.7 between SPPBrmp and SPPBt] for mobility disability). This suggests that any of the muscle power indexes could be used interchangeably for identifying older adults at risk, with the choice depending more on feasibility or data availability than on meaningful differences in predictive value.

Despite some sex- and index-specific associations, AUC results indicate that SPPB indexes perform similarly in identifying risk for mobility disability and death. This may reflect methodological differences. Cox models include time, while ROC curves assess binary outcomes at a set point. Whether higher AUCs would emerge at specific follow-up times (e.g., 12 months) remains unclear, as event numbers were too limited for further analysis.

The present study is not free of limitations. First, we examined the risk of experiencing negative events among older adults with mobility limitations, using different SPPB indexes. The possibility that SPPB scores are not the best predictor of adverse outcomes in this population exists and should be taken into consideration in interpreting the results. Indeed, muscle power and strength are substantially reduced in individuals with mobility limitations [65], which might produce considerable homogeneity in the variation of these physical capacities over time, hindering the identification of those at higher risk. Second, disability was operationalized according to the capacity to perform a gait-based test; however, associations between muscle power and the capacity to perform basic and instrumental activities of daily living still need to be examined. Third, only relatively few men were included. Fourth, the low mortality rate in SPRINTT limited consistent analyses. Fifth, longer follow-up may be needed to capture enough events for robust conclusions. Sixth, although analyses were stratified by sex, formal interaction terms (e.g., SPPB × sex) were not included in the Cox regression models. This decision was based on the low incidence of some outcomes, particularly mortality, which limited statistical power and the reliability of interaction analyses. As a result, potential effect modification by sex could not be formally assessed. Future studies with larger sample sizes and higher event rates should explore potential sex interactions to better understand whether physical performance predicts adverse outcomes differently in men and women.

## 5 Conclusions

The main findings of the present study indicate that SPPB and its derivatives are significantly associated with negative outcomes in older adults with mobility limitations. Indeed, all SPPB indexes were significantly associated with mobility disability in both sexes. However, sex-specific associations were also observed, as SPPB indexes were significantly associated with hospitalization and death in women, but not in men. When comparisons among indexes were made to identify the best-fitting model, AIC results indicated that SPPBrmp provided the best fit for mobility disability in both sexes and for death in women. In contrast, SPPBsmp exhibited the lowest AIC value for hospitalization. AUC results suggest that SPPB indexes were effective in identifying older adults with mobility limitations at higher risk of experiencing disability and death, but not hospitalization.

## Clinical trial registration

ClinicalTrials.gov identifier: NCT02582138 (registration date: 2015-10-08)

## CRediT authorship contribution statement

Conceptualization: HJCJ, AAB, EM; Methodology: HJCJ, AAB, FL, EM; Formal analysis and investigation: HJCJ, AAB, LRM, FL, EM; Writing - original draft preparation: HJCJ, AAB, LRM, FL, EM; Writing - review and editing: HJCJ, AAB, LRM, FL, EM.

## Declaration of Generative AI and AI-assisted technologies in the writing process

No Generative AI or AI -assisted technologies were used in the writing process.

## Funding

This research did not receive external funding.

## Data availability statement

Raw data files are available from Prof. Marzetti upon reasonable request.

## Declaration of competing interest

The authors declare that they have no known competing financial interests or personal relationships that could have appeared to influence the work reported in this paper.
