# Supplementary material for: Comparison of muscle strength and power in the short physical performance battery for predicting negative outcomes in older adults with mobility limitations
Source: J Nutr Health Aging. 2025 Jul 15;29(9):100631. doi: 10.1016/j.jnha.2025.100631 (PMC12284505; doi:10.1016/j.jnha.2025.100631)
Supplement: Supplementary file 1 [file mmc1.docx]

| **Supplementary Table. Sex-specific quartiles of muscle power parameters** | | | | |
| --- | --- | --- | --- | --- |
|  | **Absolute muscle power** | **Relative muscle power** | **Allometric muscle power** | **Specific muscle power** |
| *Men* | | | | |
| 1^st^ quartile (Q1) | ≤114.7 | ≤1.60 | ≤43.6 | ≤6.0 |
| 2^nd^ quartile (Q2) | 114.8−152.1 | 1.61−1.91 | 43.7−54.8 | 6.0−7.3 |
| 3^rd^ quartile (Q3) | 152.2−191.1 | 1.91−2.25 | 54.9−66.5 | 7.4−8.8 |
| 4^th^ quartile (Q4) | ≥191.2 | ≥2.26 | ≥66.6 | ≥8.9 |
| *Women* | | | | |
| 1^st^ quartile (Q1) | ≤78.5 | ≤1.27 | ≤ 33.8 | ≤5.6 |
| 2^nd^ quartile (Q2) | 78.6−101.6 | 1.28−1.57 | 33.9−42.7 | 5.7−6.9 |
| 3^rd^ quartile (Q3) | 101.7−125.9 | 1.58−1.85 | 42.8−53.4 | 7.0−8.4 |
| 4^th^ quartile (Q4) | ≥126.9 | ≥1.86 | ≥ 53.5 | ≥8.5 |
